# Spatiotemporal Dynamics of the Relative Abundance of Soil Nutrient‐Degrading Enzyme‐Encoding Genes Across Continental US Ecoregions

**DOI:** 10.1002/ece3.73869

**Published:** 2026-06-12

**Authors:** Chang Gyo Jung, Sagar Gautam, Yang Song, Kunal Poorey, Umakant Mishra

**Affiliations:** ^1^ Biomaterials & Biomanufacturing Sandia National Laboratories Livermore California USA; ^2^ Hydrology and Atmospheric Science University of Arizona Tucson Arizona USA; ^3^ Systems Biology Sandia National Laboratories Livermore California USA

**Keywords:** extracellular enzyme‐encoding genes, machine learning prediction, soil metagenomics, soil nutrient‐degrading enzyme (C, N, P)

## Abstract

Understanding the spatiotemporal patterns in the relative abundance of soil extracellular enzyme‐encoding genes is critical for predicting microbial responses to environmental change and their potential role in nutrient cycling. Yet, integrating novel metagenomic observations with spatiotemporal environmental gradients to infer regional patterns and future trajectories has remained unclear. To address this gap, we applied a machine learning (ML) approach, integrating soil metagenomic data with environmental variables—soil properties, topography, vegetation, and climate—to predict the relative abundance of enzyme‐encoding genes for soil carbon (C), nitrogen (N), and phosphorus (P) across surface soils of the continental United States. We assessed potential responses under future emission scenarios (SSP2‐4.5 and SSP5‐8.5) by comparing a baseline (1985–2014) to a future period (2071–2100). The ML model explained 57%–63% of baseline variation. Precipitation was identified as the most influential factor for the relative abundance of C‐ and N‐degrading enzyme‐encoding genes, while slope length, representing horizontal distance that water can travel downslope, was the primary driver for P‐degrading enzyme‐encoding genes abundance. Projections revealed spatially heterogeneous shifts across continental US ecoregions: the relative abundance of C‐ and N‐degrading enzyme‐encoding genes decreased in wetter ecoregions and increased in drier ecoregions under future climate, while P‐degrading enzyme‐encoding genes abundance decreased significantly in semiarid and Mediterranean ecoregions. This study demonstrates the utility of metagenomic data for mapping soil genetic potential and predicting its regional response to environmental change, to inform ecosystem management strategies.

## Graphical abstract

This study integrates large‐scale soil metagenomic data with environmental variables to map and model the relative abundance of nutrient‐degrading enzyme‐encoding genes across US ecoregions. Machine‐learning predictions reveal strong environmental controls and spatial heterogeneity in microbial functional potential, with notable shifts projected under future climate scenarios. These findings demonstrate how metagenomic data can be used to assess and forecast ecosystem‐scale nutrient cycling responses to environmental change.

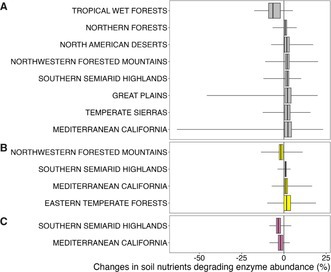

## 1 Introduction

Microbial extracellular enzymes are key to understanding how soil microbes respond to environmental conditions as they mediate nutrient acquisition and turnover of soil nutrients (Baldrian 2014; Dove et al. 2020; Wang et al. 2023). Since these enzymes catalyze key steps in the carbon (C), nitrogen (N), and phosphorus (P) cycles, it is important to quantify them for better understanding soil nutrient cycles that maintain ecosystem functions (Fanin et al. 2022). For C cycling, cellulases and ligninases depolymerize C‐rich substrates; for N cycling, peptidases and chitinases release amino acids and ammonium from proteins and chitin; and for P cycling, phosphatases and nucleases hydrolyze organic P to phosphate (Séneca et al. 2021; Wu et al. 2022; Zhang et al. 2026). The spatial distribution of these enzymes is shaped by gradients in moisture, temperature, and pH, and further modulated by topography, soil properties, vegetation, and weather, as their spatial patterns reflect environmental heterogeneity across ecoregions (Baldrian 2014; Gomez et al. 2020).

Accurate interpretation of microbial responses and ecosystem functioning requires explicitly accounting for spatial heterogeneity; otherwise, estimates of organic‐matter decomposition and nutrient cycling can be biased (Khurana et al. 2022). Spatial heterogeneity arises from (i) topography, which modulates drainage, erosion, moisture, and nutrients redistribution; (ii) soil properties (e.g., pH and temperature) that regulate enzyme catalytic rates; (iii) soil texture, which influences aeration and water retention to shape the reaction environment; and (iv) vegetation and weather conditions, which further modulate enzyme patterns (Allison et al. 2011; Burns et al. 2013; Guasconi et al. 2023; Liu et al. 2020). Since these factors are highly variable, single point measurements cannot accurately represent broader landscapes, making larger‐scale assessments necessary to fully evaluate functional potential across landscapes.

Direct measurements of C, N, and P cycling processes provide the most accurate representation of ecosystem functioning, such as CO_2_ respiration and litter decomposition for C cycling, net N mineralization and nitrification for N cycling, and phosphate turnover for P cycling (Brödlin et al. 2019; Liu et al. 2025; Mooshammer et al. 2017; Owen et al. 2003). However, these realized process rates do not always correspond directly to enzyme activities or abundances because most enzyme activity measurements represent potential rates generated under ideal assay conditions, making them not directly comparable to field‐based assessments (Lane et al. 2022). Despite these limitations, previous studies have attempted to link the abundance of enzyme‐encoding genes to nutrient cycling processes. For example, cellulase gene abundance across agricultural systems has been used to demonstrate how management practices shape microbial genetic potential (Choi et al. 2018). Larger‐scale analyses have revealed contrasting effects on N and P metabolic processes (Poeplau et al. 2016), as well as region‐specific patterns in the relative abundance of fungi and bacteria (Yu et al. 2022). In addition, catchment‐scale distributions of C‐, N‐, and P‐cycling enzyme activities have been documented (Keller et al. 2023). Collectively, these findings suggest that strong biogeographical patterns influence microbial functional potential.

Extracellular enzyme activity in soils is measured using laboratory enzyme assays under controlled environments. These assays quantify potential reaction rates in the ideal condition for specific extracellular enzyme (Sorouri and Allison 2022; Xu et al. 2021). Extending enzyme‐level measurements to ecosystem responses is challenging, but large‐scale assessments of enzyme‐encoding genes, for example, GeoChip and batches of quantitative PCR (qPCR), and shotgun metagenomics can quantify the functional potential of microbial communities; in some cases, this information facilitates the linkage of enzyme activities to ecosystem biogeochemical processes like C and N cycling (He et al. 2007; Ouyang et al. 2018; Ouyang and Norton Jeanette 2020). Moreover, metagenomic approaches can characterize functional genes across pathway and reveal environmental controls on microbial functional potential (Bai et al. 2025; Mushinski et al. 2021; Wang et al. 2022).

Among these techniques, the metagenomic approach stands out for its ability to provide a comprehensive snapshot of the functional potential of soil ecosystems. Indeed, metagenome‐derived abundance of enzyme‐encoding genes could be a strong proxy to understand levels of enzyme activity or microbial functional potentials (Daniel 2005; Fierer et al. 2012; Manoharan et al. 2017). By identifying and quantifying the abundance of enzyme‐encoding genes in metagenomes, it allows for the formulation of hypotheses about how soil ecosystems respond to specific environmental conditions (Sokol et al. 2022). This is particularly valuable as it overcomes the limitations of traditional laboratory‐cultured microbial experiments, which cannot fully replicate the complexity of natural soil environments (Ejaz et al. 2024; Sieradzki et al. 2023). Gene abundance in metagenomes serves as a proxy for the potential to encode the corresponding enzyme and is used to assess how potential enzyme activities in soil respond to different environmental conditions (Anthony et al. 2024; Garg et al. 2024; Trivedi et al. 2016).

Furthermore, the integration of data from multiple study sites holds significant promise for generating new insights into soil enzyme dynamics. Synthesizing such datasets can help uncover broad patterns and relationships that are not apparent in single‐site studies (Ma et al. 2023; Mantri et al. 2021). However, despite the potential of these approaches, research on the spatial and temporal variations in soil enzyme abundance remains relatively scarce. A recent continental‐scale study identified specific edaphic (e.g., soil C, C/N ratio, and texture), climate (e.g., precipitation and temperature), and vegetation (net primary productivity) factors regulating the spatial distribution of enzyme functional classes related to soil C, N, and P cycling (Fan et al. 2025). These environmental factors influence microbial resource optimization strategies across different ecosystems, determining how microbial communities allocate metabolic investment toward nutrient acquisition and decomposition processes under varying environmental constraints. Understanding these variations in microbial functional potential is therefore critical for advancing our mechanistic understanding of soil biogeochemical processes and their implications for ecosystem functioning. Expanding the scope of studies across diverse spatial and temporal scales could lead to breakthroughs in predicting soil ecosystem responses to environmental changes, improving the robustness of ecosystem models.

This study quantifies the relative abundance of enzyme‐encoding genes involved in soil C, N, and P decomposition processes by leveraging publicly available metagenomic datasets from the Integrated Microbial Genomes & Microbiomes database (IMG/M; Chen et al. 2021). In this study, we address two key research questions: (1) Which environmental factors control the spatial distribution of enzyme‐encoding genes? and (2) How may these enzyme‐encoding genes shift under future climate scenarios? To answer these questions, we used a random forest machine learning model that integrates metagenomic observations with environmental covariates, including topographic features, soil properties, and climatic variables. This integration enables the development of spatially explicit maps representing current gene abundance as a proxy for microbial functional potential across diverse landscapes. Future projections were generated using future climate data from the Coupled Model Intercomparison Project Phase Six (CMIP6) under two Shared Socioeconomic Pathways (SSP2‐4.5 and SSP5‐8.5) for the period 2071–2100. We hypothesize that specific ecoregions will exhibit significant sensitivity to environmental change, resulting in higher or lower gene abundance compared to baseline conditions. By linking current distributions with future climate projections, this approach demonstrates the potential of metagenomic data to predict shifts in soil biogeochemical processes under changing environmental conditions.

## 2 Materials and Methods

### 2.1 Metagenomic Data

To determine the relative abundance of enzyme‐encoding genes, we used metagenomic annotation tables (Pfam annotations) from the Joint Genome Institute (JGI) Integrated Microbial Genomes & Microbiomes (IMG/M) database (Chen et al. 2021). These tables provide a robust framework for profiling potential protein families identified from sequence reads (Mistry et al. 2021). Since our study focuses on surface soil biogeochemistry, we filtered the database to exclusively select datasets explicitly sourced from terrestrial topsoils (0–30 cm depth) within the Continental United States (CONUS). This approach ensures our targets are directly linked to relevant soil biogeochemical processes, supported by established strong relationships between functional gene abundance and enzyme activities (Ouyang et al. 2018; Trivedi et al. 2016).

Applying these criteria yielded a total of 2884 metagenomic datasets from 803 unique sampling locations (Table S1). To assess the genetic potential for nutrient cycling across CONUS ecoregions, we quantified the relative abundance of enzyme‐encoding genes involved in the decomposition of C, N, and P, grouping them into three corresponding functional classes. For C‐degrading enzyme, we utilized the Carbohydrate‐Active enZYmes (CAZy) database, which includes five major enzyme categories responsible for complex carbohydrate breakdown: Glycoside Hydrolase (GH), Glycosyltransferase (GT), Polysaccharide Lyase (PL), Carbohydrate Esterase (CE), and Carbohydrate‐Binding Module (CBM) (Drula et al. 2022). We used the R package of ‘*PFAM.db*’ for mapping such protein families associated with specific Pfam accession numbers (Carlson et al. 2023).

For N‐degrading enzyme, we targeted enzyme‐encoding genes that depolymerize proteinaceous and other nitrogenous organic matter (Séneca et al. 2021). For P‐degrading enzyme, we selected hydrolases associated with the degradation of phospholipids (e.g., phospholipases) and nucleotides (e.g., nucleases), which are essential for P cycling in soil ecosystems (Bashir Ahmed Siddique et al. 2023; Pant and Warman 2000).

Finally, to ensure comparability across the diverse metagenomic datasets with varying sequencing depths, we calculated the relative abundance of these targeted enzyme‐encoding genes. Specifically, the read counts for the selected enzyme‐encoding genes in each sample were normalized against the total number of Pfam annotations detected in that same sample. This normalization minimized bias from sequencing depth and provided a consistent basis for assessing the spatial distributions of key nutrient‐degrading functional genes across ecoregions. Detailed information of soil nutrient‐degrading protein families (Pfam accession numbers) is provided in Table S2.

### 2.2 Environmental Data

We compiled a comprehensive set of environmental variables relevant to soil ecological processes along with spatial coordinates (latitude and longitude) (Gautam et al. 2022). These included soil properties (e.g., soil pH, soil organic carbon, soil order, soil drainage class, soil temperature/moisture regime, aridity index and hydrology type), topographic attributes (e.g., slope height, slope length, slope gradient, surface geology, flatness index, altitude channel, aspect, elevation, and topographic wetness index), vegetation (e.g., potential vegetation cover, land cover type, net primary productivity, and ecoregion types) and climatic factors (e.g., maximum/minimum temperature and annual precipitation). For the climatic baseline, we used a 30‐year reference period (1985–2014) to calculate climatological mean conditions. Although some metagenomic samples were collected outside this timeframe, the use of long‐term climate normals provide a consistent environmental framework for relating present‐day metagenomic observations to prevailing climatic conditions and enables a more robust comparison with projected future climates for the period 2071–2100. This approach is consistent with standard climatological practices and facilitates the assessment of potential shifts in relative abundance of enzyme‐encoding genes under future scenarios. As for net primary productivity and soil organic carbon, we selected data from 2014, marking the end of the baseline period. Since future climate conditions are also likely to alter net primary productivity, we used net primary productivity at 2100 for the future projection. To ensure spatial consistency across datasets, every environmental covariate layer was resampled to a 100 m resolution (detailed descriptions of all data sources are provided in Table S3). Since approximately one third of total dataset originated from shared geographic coordinates (e.g., repeated sampling at the same site), we extracted and assigned identical environmental covariate values to these co‐located datasets.

### 2.3 Geospatial Modeling

To perform geospatial modeling, we extracted environmental data from all covariate map layers and integrated them with the relative abundance of C‐, N‐, and P‐degrading enzyme‐encoding genes. We used a random forest machine learning (ML) algorithm to generate spatially explicit maps of potential enzyme abundance for each functional category. Random forest is an ensemble learning method that aggregates outputs from numerous decision trees, making it particularly robust against non‐normal data distributions and multicollinearity among predictive variables (Breiman 2001; Pichler and Hartig 2023). This approach has been widely established for predicting the spatial distributions of soil organic carbon (SOC) (Gautam et al. 2022; Mishra et al. 2022). To optimize the models for each enzyme category, we randomly split the dataset allocating 70% of the samples for training and hyperparameters tuning, reserving 30% for independent validation. During training, we applied a 10‐fold cross‐validation with two repetitions. The model exhibiting the lowest RMSE and highest *R* ^2^ was selected for spatial prediction across CONUS. The final optimized hyperparameters for the C, N, and P models used 7, 5, and 5 randomly selected predictors (m_try_), respectively with minimal node sizes (min_n) of 10, 13, and 23, all using 500 trees. The final projections were then validated against the remaining 30% of samples.

To evaluate potential responses to environmental change, we compared baseline enzyme abundance estimates with projections under two Shared Socioeconomic Pathway Scenarios (SSP 2–4.5 and SSP5‐8.5). Future projections were generated by applying the validated baseline ML model to future climate and NPP datasets from CMIP6 model outputs, that is, BCC‐CSM2‐MR, CanESM5‐CanOE, UKESM1‐0‐LL, and CESM2 (Danabasoglu 2019; Good et al. 2019; Swart et al. 2019; Xin et al. 2018). We calculated the difference between baseline and future relative abundances of enzyme‐encoding genes, and stratified the results by Level 1 ecoregions (Omernik and Griffith 2014) to analyze ecoregion‐specific responses (Figure S1). This approach provided critical insights into how the genetic potential for soil nutrients degradation may shift under varying climatic conditions and vegetation productivity. All data processing, modeling, and visualization were performed in R version 4.3.2 (R Core Team 2023) with the tidymodels, terra and ggplot2 packages (Hijmans 2024; Kuhn and Wickham 2020; Wickham 2016).

## 3 Results

### 3.1 Prediction of Relative Abundance of Enzyme‐Encoding Genes Using ML Models

**FIGURE 1:**
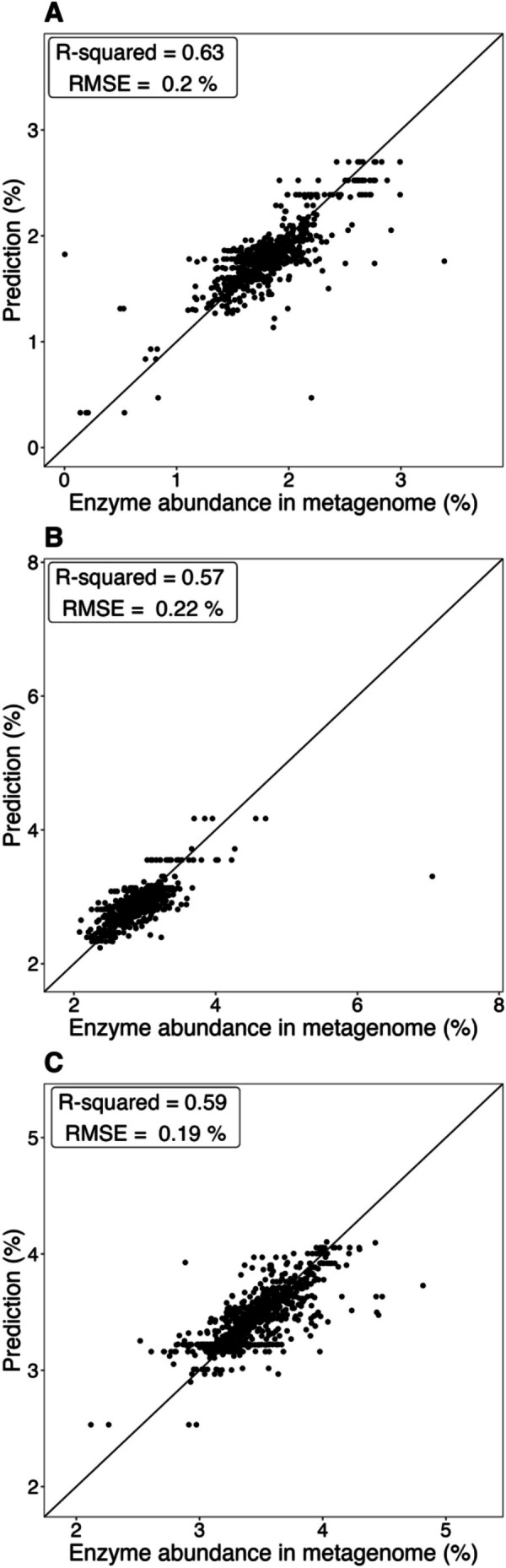
Validation accuracy of the machine learning models for relative abundance of C‐ (A), N‐ (B), and P‐degrading enzyme‐encoding genes (C). RMSE is root mean square error.

**FIGURE 2:**
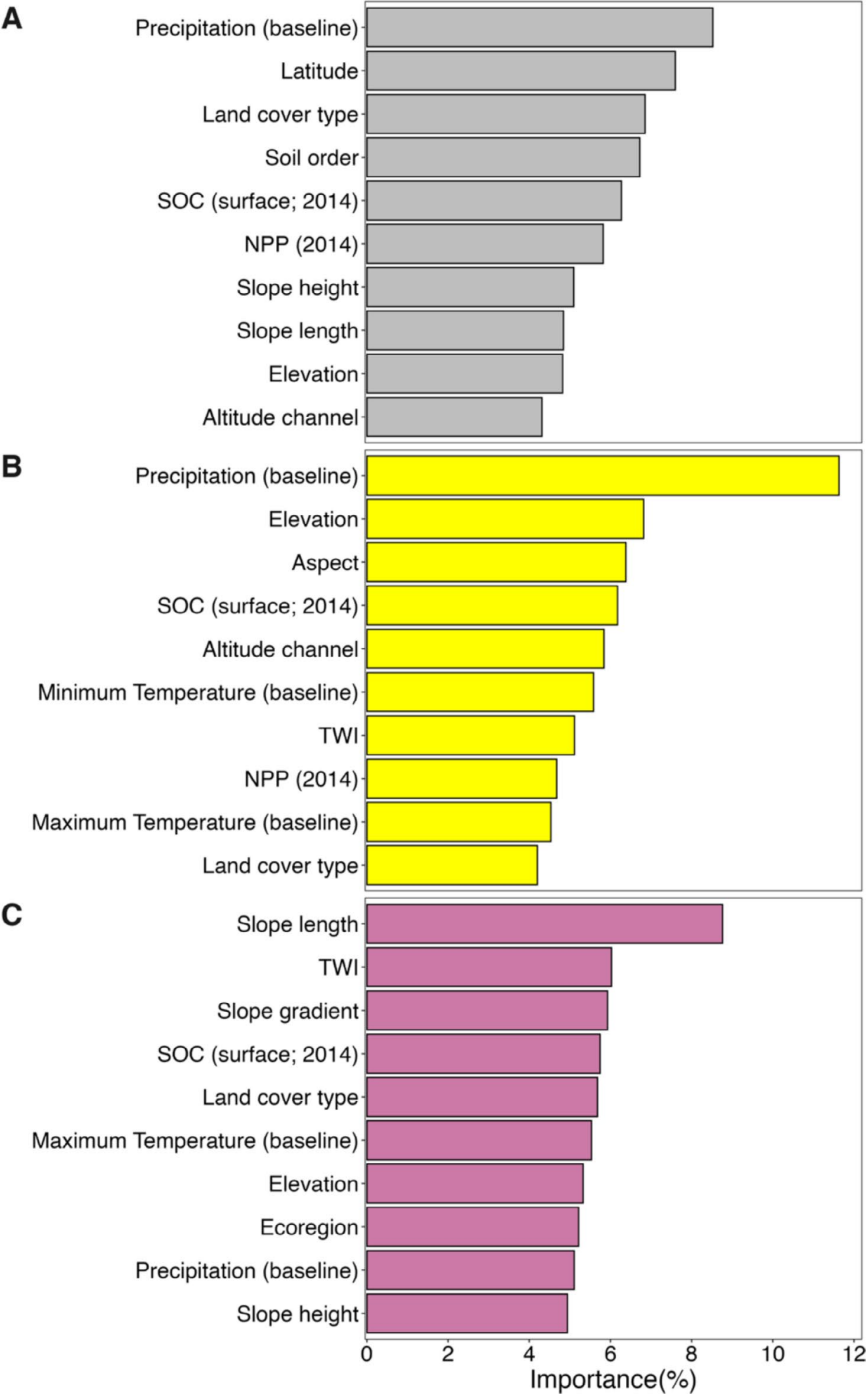
Top 10 most important environmental factors in predicting the relative abundance of enzyme‐encoding genes in the continental United States under baseline environmental conditions (1985–2014). Bars indicate each predictor's percent share of total impurity‐based importance. Gray (A; C‐degrading enzymes), yellow (B; N‐degrading enzymes), and pink (C; P‐degrading enzymes) indicate different enzyme categories. TWI is topographic wetness index, SOC is soil organic carbon, and NPP is net primary productivity.

The ML model, which integrated environmental factors such as soil properties, topography, vegetation and climate variables, successfully predicted the relative abundance of C‐, N‐ and P‐degrading enzyme‐encoding genes, accounting for 57%–63% of the observed variations and having RMSE ranging from 0.19% to 0.22% (Figure 1). Among the environmental factors used, baseline precipitation (30‐year mean) was the primary predictor for relative abundance of C‐ (12%) and N‐degrading enzyme‐encoding genes (9%), while slope length (9%) was identified as the primary determinant for the relative abundance of P‐degrading enzyme‐encoding genes (Figure 2). In addition, drainage (0.4% and 0.2% for relative abundance of C‐ and N‐degrading enzyme‐encoding genes, respectively) and hydrology type (0.8%; relative abundance of P‐degrading enzyme‐encoding genes) contributed least to the model (Figure S2).

### 3.2 Baseline Relative Abundance of Enzyme‐Encoding Genes Across US Ecoregions

**FIGURE 3:**
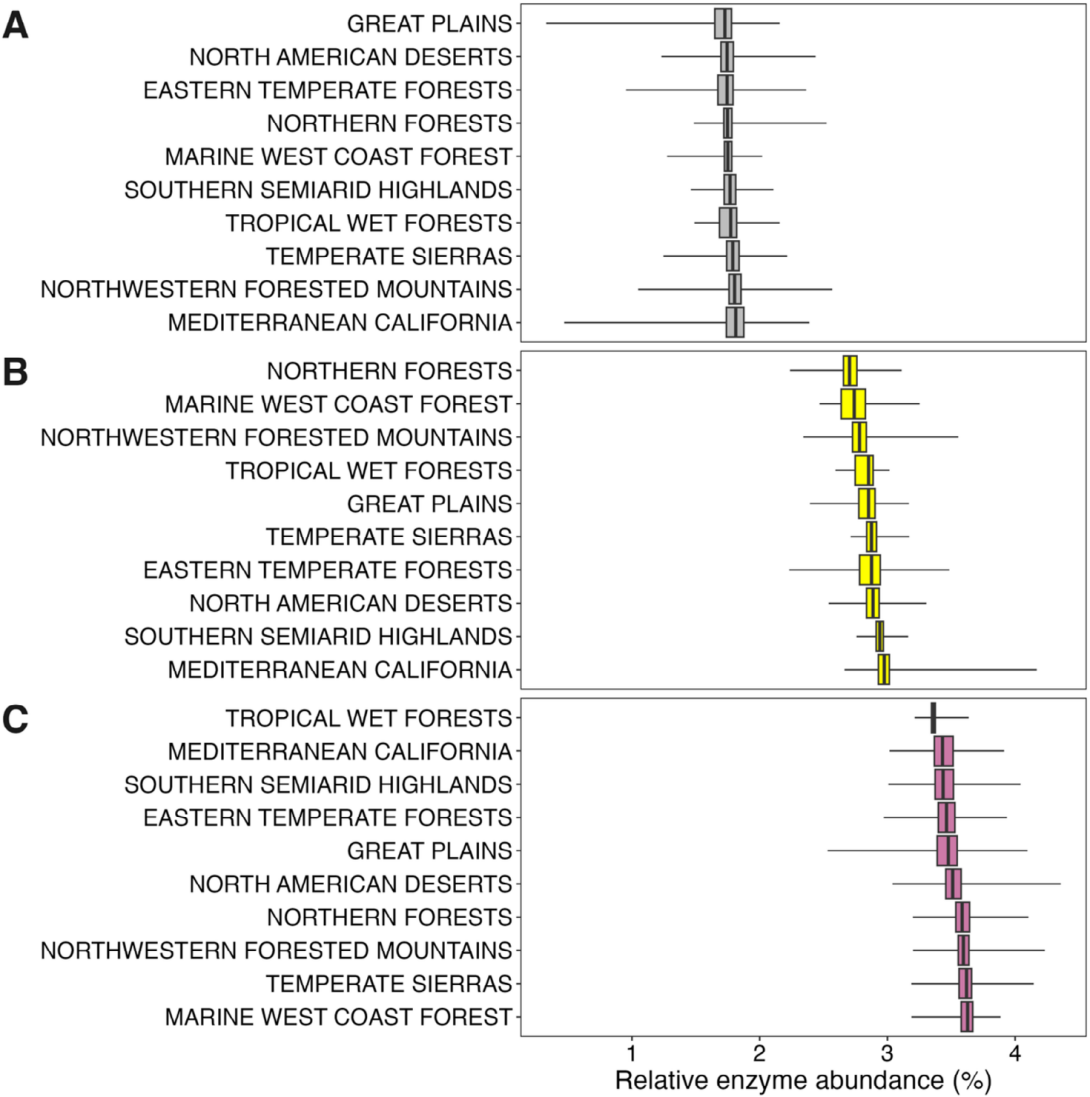
Relative abundance of C‐ (A; gray), N‐ (B; yellow), and P‐degrading (C; pink) enzymes‐encoding genes across continental UnS ecoregions (Level 1; Omernik and Griffith 2014).

Under baseline conditions, the relative abundance of C‐, N‐, and P‐degrading enzyme‐encoding genes exhibited spatial heterogeneities across ecoregions (Figure S3). For example, Great Plains and northern forests ecoregions showed the lowest relative abundance of C‐ and N‐degrading enzyme‐encoding genes (1.72% and 2.70%, respectively) (Figure 3). In contrast, the Mediterranean California ecoregion exhibited the highest relative abundances, with 1.80% for C‐degrading enzyme‐encoding genes and 3.00% for N‐degrading enzyme‐encoding genes. Regarding P‐degrading enzyme‐encoding genes, the Tropical Wet Forests had the lowest relative abundance at 3.36%, while the marine west coast forest had the highest level at 3.63%.

### 3.3 Relative Abundance of Enzyme‐Encoding Genes Across US Ecoregions Under Future Climate

**FIGURE 4:**
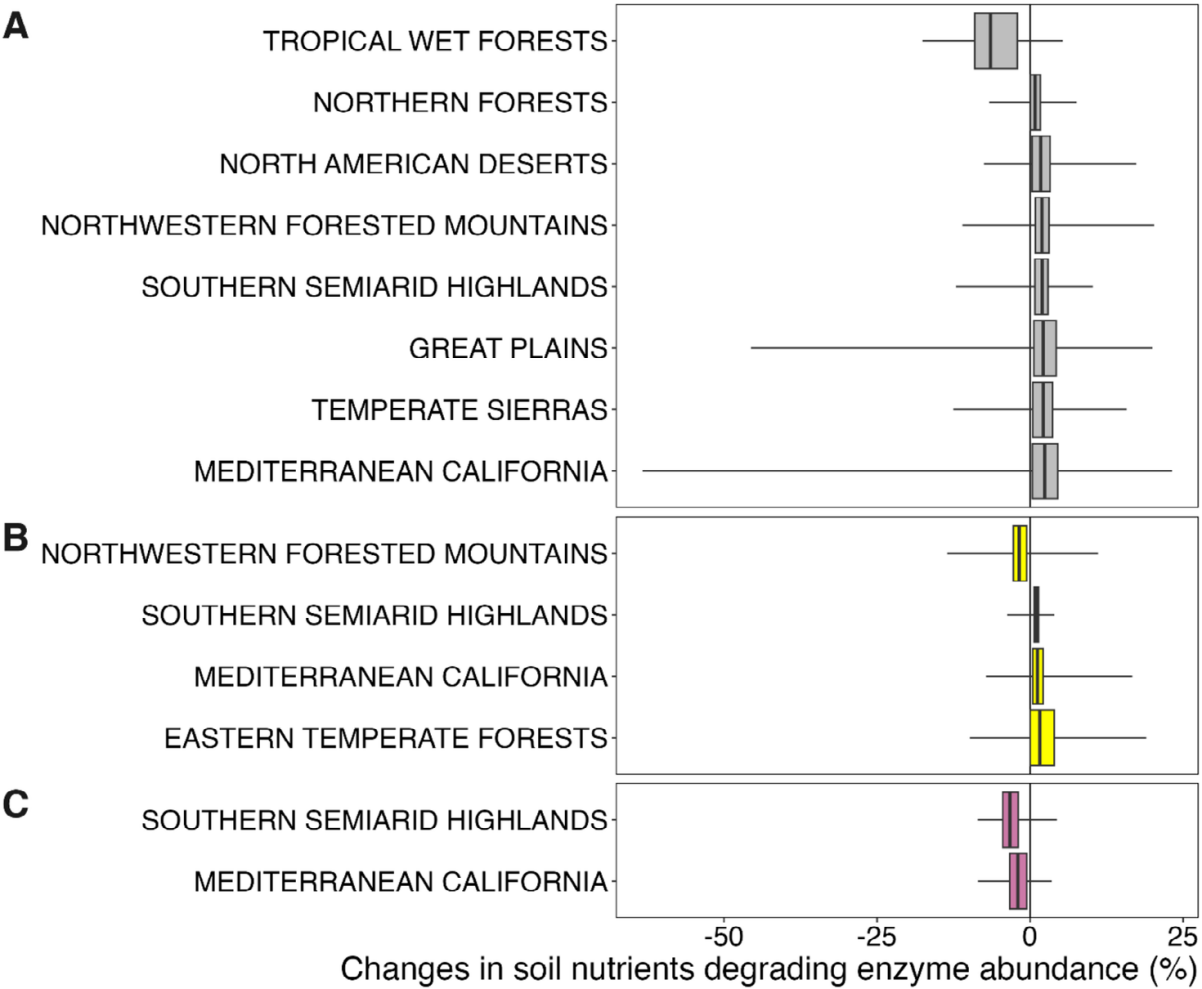
Changes in the relative abundance of C‐ (A; gray), N‐ (B; yellow), and P‐degrading (C; pink) enzymes‐encoding genes under the SSP5‐8.5 scenario across continental US ecoregions.

Using the established ML models and climate projections, we mapped future relative abundance of C‐, N‐, and P‐degrading enzyme‐encoding genes (Figure S4). Compared to baseline conditions, the SSP5‐8.5 scenario projected notable shifts in these genetic potentials (Figures S5 and S6). Specifically, the relative abundance of C‐degrading enzyme‐encoding genes significantly increased (0.82%–2.48% relative to the baseline level) across seven ecoregions of 10 ecoregions: Northern Forests, North American Deserts, Northwestern Forested Mountains, Southern Semiarid Highlands, Great Plains, Temperate Sierras, and Mediterranean California. However, this relative abundance significantly decreased (−6.45%) in Tropical Wet Forests (Figure 4A). For N‐degrading enzyme‐encoding genes, three ecoregions (Southern Semiarid Highlands, Mediterranean California, and Eastern Temperate) showed significant increases in relative abundance ranging from 1.03% to 1.60%, whereas Northwestern Forested Mountains showed a decrease of −1.75% (Figure 4B). The relative abundance of P‐degrading enzyme‐encoding genes is predicted to significantly decrease in Southern Semiarid Highlands (−3.28%) and Mediterranean California (−1.99%) (Figure 4C).

## 4 Discussion

This study leveraged observed metagenomic datasets from the IMG/M database to train machine learning (ML) algorithms, successfully mapping the heterogeneous spatial distributions of C‐, N‐, and P‐degrading enzyme‐encoding genes across the continental United States (Figures S3 and S4). We found that environmental covariates are essential for predicting these patterns (Figure 2, Figure S4), and our approach addressed our research questions. Firstly, to identify the main environmental factors controlling the spatial distribution of these genes, we found that precipitation is the most critical factor influencing the relative abundances of C‐ and N‐degrading enzyme‐encoding genes, while slope length emerged as the key variable for P‐degrading enzyme‐encoding genes. Second, to project how this functional potential might shift under future emission scenarios, our models supported our initial hypothesis by revealing that specific ecoregions were sensitive to environmental change, showing distinct increases or decreases in relative abundance compared to baseline conditions. These results provide critical insights into how the relative abundance of genes encoding microbial enzymes can be leveraged to analyze spatial patterns for biogeochemical cycling and predict their responses to changing environmental conditions.

### 4.1 Importance of Environmental Factors

Soil temperature and moisture are well‐established factors influencing the decomposition of soil nutrients (Li et al. 2024; Sinsabaugh et al. 2016; Zhou et al. 2020). In general, with increases in soil temperature, positive effects of temperature on soil C decomposition exponentially increase until they saturate at the maximum enzymatic capacity while moisture sensitivity saturates more quickly and decreases under excessively wet conditions. Furthermore, these two factors often interact, for example, water frequently becomes a limiting factor as temperature increases (Liu et al. 2024; Pallandt et al. 2022; Sierra et al. 2015; Wu et al. 2022). Similarly, nitrification, denitrification rates, and phosphatase activities depend on coupled soil temperature and moisture dynamics (Pan et al. 2022). Consistent with this understanding, our analysis revealed precipitation as a primary driver of the relative abundances of C‐ and N‐degradation enzymes‐encoding genes whereas it showed lower importance for P‐degrading enzyme‐encoding genes (Figure 2). This aligns with results that P cycling is more strongly associated with anthropogenic P and N deposition than precipitation variability (Abbasi et al. 2020; Shaw and Cleveland 2020; Yue et al. 2018).

In addition to the contributions of temperature and moisture, elevation and land cover type are common important factors regulating the relative abundances of soil nutrient‐degrading enzyme‐encoding genes. Topography modulates microclimates and creates diverse microhabitats (Guasconi et al. 2023; Liu et al. 2020) that directly regulate microbial activities and interact with soil properties like pH and soil texture (Aciego Pietri and Brookes 2008; Malik et al. 2018). Changes in land cover type alter litter quality and water table depth, leading to shifts in potential microbial activities and greenhouse gas emissions (Evans et al. 2021; Peña‐Peña and Irmler 2022). Another important finding is the critical role of SOC in driving soil nutrient decomposition (Figure 2). Since organic C substrate is the main source for the microbial activities, SOC availability fundamentally limits microbial genetic potential (Soong et al. 2018). Additionally, NPP and latitude played important roles in the C‐ and N‐degrading enzyme models, respectively (Figure 2). Such patterns likely reflect latitudinal controls on soil extracellular enzyme activities driven by radiation‐mediated environmental changes (Jian et al. 2021) and the indirect effects of vegetation characteristics on soil nutrient inputs (Guasconi et al. 2023). Together, these variables accounted for substantial proportions of the explained variance in the relative abundance of C‐, N‐ and P‐degrading enzyme‐encoding genes (Figure 2, Figure S2).

### 4.2 Relative Abundance of Enzyme‐Encoding Genes Under Future Climate

The spatial heterogeneity of individual environmental variables and temporal variability of climatic variables contributed to significant differences in the relative abundance of enzyme‐encoding genes across ecoregions (Figures 3 and 4). This is expected, as ecoregions are defined by the integration of physical and biological factors that align with the environmental drivers identified in our study (Commission for Environmental Cooperation 1997; Hughes and Omernik 1999; Table S3).

Notably, the future climate projections (2071–2100; SSP5‐8.5) are expected to alter these baseline biogeographical patterns (1985–2014) across ecoregions (Figure 4, Figures S5 and S6). Our results indicate significant increases in the relative abundance of C‐degrading enzyme‐encoding genes across seven of the ecoregions: Northern Forests, North American Deserts, Northwestern Forested Mountains, Southern Semiarid Highlands, Great Plains, Temperate Sierras, and Mediterranean California. These ecoregions are anticipated to face intensified drought and aridification (Balik et al. 2024). Such responses are influenced by the complex interactions between water stress, nutrient availability, and temperature. Moderate drought and nutrient‐limited conditions may stimulate microbial communities to increase their genetic potential for enzyme synthesis to acquire scarce resources (Bouskill et al. 2016; Tariq et al. 2024). In contrast, a significant decreasing trend in the relative abundance of C‐degrading enzyme‐encoding genes was observed in Tropical Wet Forests (Figure 4A). This opposing trend is likely due to that ecoregion's distinct ecological dynamics and climate sensitivities. Given higher baseline moisture and distinct nutrient regimes, rising temperatures in these ecosystems may push beyond the optimum thermal ranges of microbial metabolism, leading to a suppression in the community's capacity (Wood et al. 2012). Moreover, projected shifts in NPP within these forests may alter C substrate availability, leading to shifts in microbial nutrient acquisition strategies rather than increases in C‐degradation potential (Koch and Kaplan 2022; Wong et al. 2024).

The relative abundance of N‐degrading enzyme‐encoding genes showed increasing trends in Southern Semiarid Highlands, Mediterranean California, and Eastern Temperate while the Northwestern Forested Mountains exhibited a decrease (Figure 4B). Changes in the relative abundance of P‐degrading enzyme‐encoding genes were particularly pronounced in Southern Semiarid Highlands and Mediterranean California, which showed significant decreases, indicating higher sensitivity to declining precipitation and increasing temperatures (Figure 4C). These findings are consistent with previous studies on the climate sensitivities of P‐degrading enzyme (Bashir Ahmed Siddique et al. 2023; Fanin et al. 2022; Wu et al. 2022), highlighting the highly variable, ecoregion‐specific responses of soil microbial communities to future environmental change.

### 4.3 Limitations and Future Directions

We show that the ML approach using metagenomic datasets can identify drivers and project future shifts, but more detailed information is needed to link gene abundance to realized decomposition rates. The decomposition of soil nutrients is governed not only by degrading enzymes but also by interactions among various enzyme groups and their activity levels, which are influenced by environmental conditions (Baldrian 2014; Renaud and Martiny 2013; Fan et al. 2025). Our approach grouped enzyme‐encoding genes by soil nutrient‐specific categories (C, N, and P), implicitly assuming uniform activity levels across enzymes. In natural environments, decomposition potential varies with enzyme composition, substrate composition, substrate quality and availability, and soil minerals interactions that can stabilize or inhibit enzymes' activities (Bouskill et al. 2016; Fanin et al. 2022; Zhou et al. 2020). Future work should integrate multi‐omics data and couple these with biogeochemical models calibrated against enzyme activities and microbial composition (Herold et al. 2020; Jansson and Hofmockel 2018). Such integrations will refine the microbial representations in biogeochemical models, improving their predictability under changing environments.

Another limitation is the spatial and temporal resolutions of various datasets used. The environmental covariates resampled at 100 m resolution may not adequately capture finer‐scale spatial heterogeneity. Addressing this limitation will require multiscale modeling that integrate molecular‐level processes (e.g., metagenomic information) with ecosystem‐level outcomes (e.g., nutrient fluxes and storage). This would provide a crucial link between the fine‐scale dynamics of soil enzyme activities and larger‐scale ecosystem dynamics.

Additionally, while this study used a comparison of 30‐years climate normal (baseline vs. future climate) to provide a robust snapshot of potential long‐term shifts in the relative abundance of enzyme‐encoding genes, it does not account for short‐term fluctuations, seasonal dynamics, or uncertainties in long‐term microbial community responses. Incorporating high‐frequency temporal data could provide a more comprehensive understanding of how enzyme‐encoding gene abundances respond to environmental covariates and environmental change over time. Despite these limitations, future research can build upon our methodological framework to enhance the predictive models and improve our understanding of the dynamics of nutrient degrading enzyme‐encoding genes in the context of environmental change.

## 5 Conclusions

Our study demonstrates the utility of soil metagenomic data for projecting potential responses of soil enzyme‐encoding genes to environmental change across the continental US ecoregions, providing a scalable approach to assess spatiotemporal variations in soil biogeochemical cycles. By integrating ML models with environmental covariates, we generated precise predictions and spatial maps regarding the relative abundance of these genes. While this approach demonstrates potential for advancing research on biogeochemical cycling, remaining limitations should be addressed in future studies. These include (1) refining substrate classifications and their specific enzymes to reflect variability in decomposition processes (Renaud and Martiny 2013), (2) using higher resolution spatial data to capture microheterogeneity, and (3) incorporating more frequent temporal datasets to capture seasonal dynamics and interannual dynamics. Future efforts should quantify how variations in the relative abundance of enzyme‐encoding genes translate to ecosystem‐scale processes (e.g., nutrient turnover, C storage and greenhouse‐gas fluxes). This may advance understanding of biogeochemical cycling and improve prediction of ecosystem responses to environmental change.

## Author Contributions

**Chang Gyo Jung:** conceptualization (lead), data curation (lead), formal analysis (lead), methodology (lead), resources (lead), software (lead), visualization (lead), writing – original draft (lead), writing – review and editing (lead). **Sagar Gautam:** visualization (supporting), writing – review and editing (supporting). **Yang Song:** formal analysis (supporting), methodology (supporting), writing – review and editing (supporting). **Kunal Poorey:** writing – review and editing (supporting). **Umakant Mishra:** conceptualization (equal), formal analysis (supporting), funding acquisition (lead), investigation (equal), methodology (equal), project administration (lead), supervision (lead), writing – review and editing (equal).

## Funding

This work was supported by Sandia National Laboratories (DE‐NA‐0003525).

## Conflicts of Interest

The authors declare no conflicts of interest.

## Supporting information


**Table S1:** Descriptions of metegenomic datasets in the Integrated Microbial Genomes & Microbiomes (IMG/M).

**Table S2:** PFAM accession numbers and their descriptions for each soil nutrient type.

**Table S3:** List of environmental variables and their descriptions.

**Figure S1:** Level 1 ecoregion map.
**Figure S2:** Importance of environmental factors in predicting soil enzyme abundance in continental United States. Gray (A; C‐degrading enzyme), yellow (B; N‐degrading enzyme) and pink colors (C; P‐degrading enzyme) indicate different enzyme categories.
**Figure S3:** Spatial maps of carbon (A), nitrogen (B), and phosphorous (C) degrading enzyme abundance in the baseline period (1985–2014).
**Figure S4:** Spatial maps of carbon, nitrogen, and phosphorous degrading enzyme abundance under the future Shared Socioeconomic Pathways (SSP) 245 (A–D) and 585 (E–H). A and E: BCC‐CSM2‐MR, B and F: CanESM5‐CanOE, C and G: UKESM1‐0‐LL, D and H: CESM2.
**Figure S5:** Spatial maps of changes (%) in carbon, nitrogen, and phosphorous degrading enzyme abundance under the future Shared Socioeconomic Pathways (SSP) 245 (A‐D) and 585 (E‐H). A and E: BCC‐CSM2‐MR, B and F: CanESM5‐CanOE, C and G: UKESM1‐0‐LL, D and H: CESM2.
**Figure S6:** Changes in carbon (A), nitrogen (B), and phosphorous (C) degrading enzyme abundance due to scenarios (SSP 245, blue color; SSP 585, orange color).

## Data Availability

All the data and codes are available at figshare (https://figshare.com/s/eb842af4b497d91529c5).
