# Supplementary material for: Spatiotemporal Dynamics of the Relative Abundance of Soil Nutrient‐Degrading Enzyme‐Encoding Genes Across Continental US Ecoregions
Source: Ecol Evol. 2026 Jun 12;16(6):e73869. doi: 10.1002/ece3.73869 (PMC13263121; doi:10.1002/ece3.73869)
Supplement: Supplementary file 4 — Figure S1: Level 1 ecoregion map. Figure S2: Importance of environmental factors in predicting soil enzyme abundance in continental United States. Gray (A; C‐degrading enzyme), yellow (B; N‐degrading enzyme) and pink colors (C; P‐degrading enzyme) indicate different enzyme categories. Figure S3: Spatial maps of carbon (A), nitrogen (B), and phosphorous (C) degrading enzyme abundance in the baseline period (1985–2014). Figure S4: Spatial maps of carbon, nitrogen, and phosphorous degrading enzyme abundance under the future Shared Socioeconomic Pathways (SSP) 245 (A–D) and 585 (E–H). A and E: BCC‐CSM2‐MR, B and F: CanESM5‐CanOE, C and G: UKESM1‐0‐LL, D and H: CESM2. Figure S5: Spatial maps of changes (%) in carbon, nitrogen, and phosphorous degrading enzyme abundance under the future Shared Socioeconomic Pathways (SSP) 245 (A‐D) and 585 (E‐H). A and E: BCC‐CSM2‐MR, B and F: CanESM5‐CanOE, C and G: UKESM1‐0‐LL, D and H: CESM2. Figure S6: Changes in carbon (A), nitrogen (B), and phosphorous (C) degrading enzyme abundance due to scenarios (SSP 245, blue color; SSP 585, orange color). [file ECE3-16-e73869-s002.docx]

**Supporting Information**

**Spatiotemporal dynamics of the relative abundance of soil nutrient-degrading enzyme-encoding genes across continental United States ecoregions**

Chang Gyo Jung^1*^, Sagar Gautam^1^, Yang Song^2^, Kunal Poorey^3^, Umakant Mishra^1^

^1^Biomaterials & Biomanufacturing, Sandia National Laboratories, Livermore, CA 94550

^2^Hydrology and Atmospheric Science, University of Arizona, Tucson, AZ, 85721

^3^Systems Biology, Sandia National Laboratories, Livermore, CA 94550

* Corresponding author: Chang Gyo Jung ([cjung@sandia.gov](mailto:cjung@sandia.gov))

The following supporting materials provide additional information on the 10 ecoregions (Figure S1) and importance of variations in gene-encoding soil nutrients (carbon; C, nitrogen; N and P; phosphorous) degrading enzymes for each machine learning (ML) model as well as figures that support the main texts (Figure S2-S6). Figure S1 shows 10-Level 1 ecoregions in continental US (CONUS). Figure S2 highlights the importance of variances for each ML model and projections of gene-encoding enzymes across CONUS (Figure S3). Figure S4 shows maps of projections under future emission scenarios based on Shared Socioeconomic Pathways (SSP), specifically, SSP 245 and 585, along with their differences compared to baseline conditions (Figures S5 and S6).


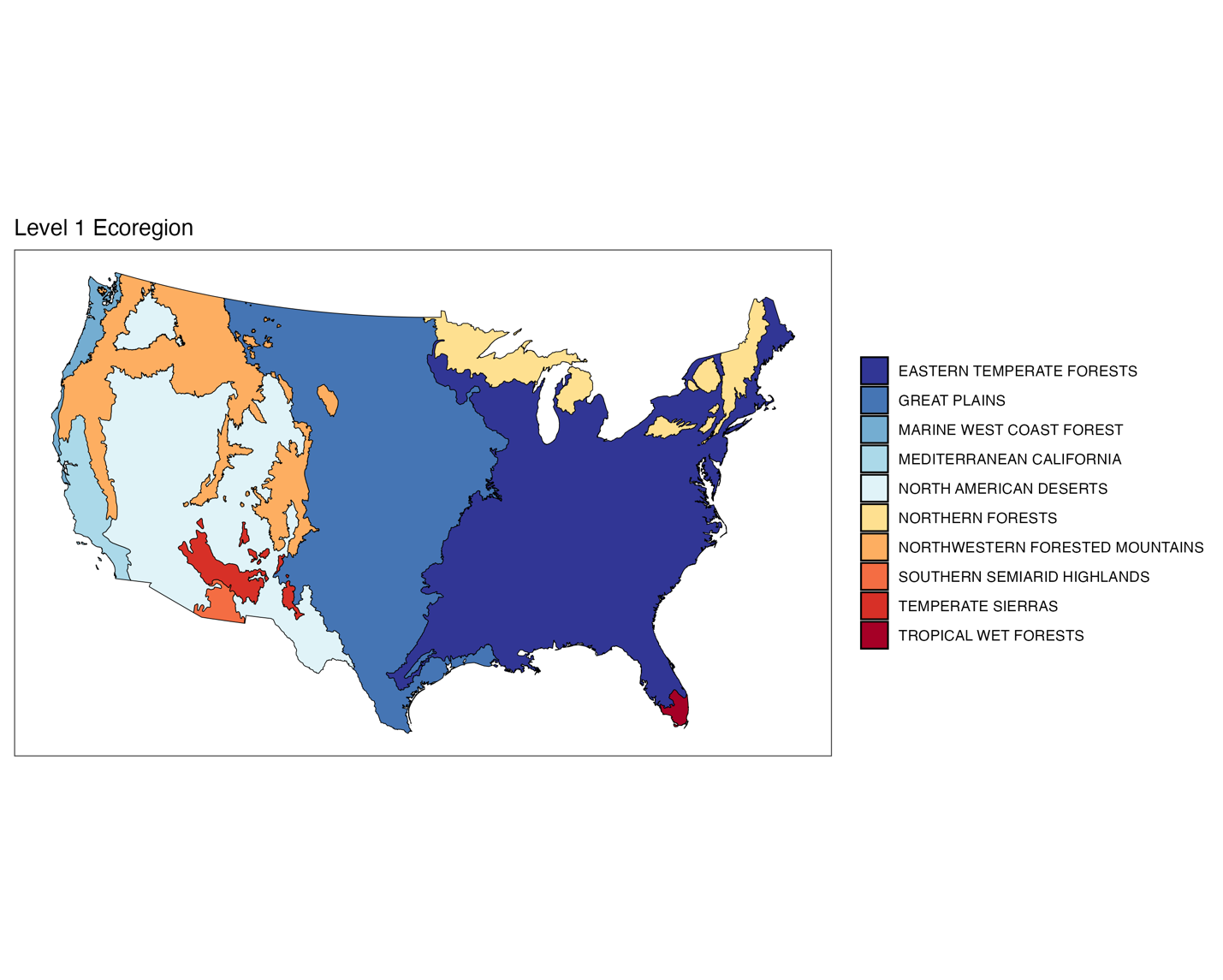


Figure S1. Level 1 ecoregion map.


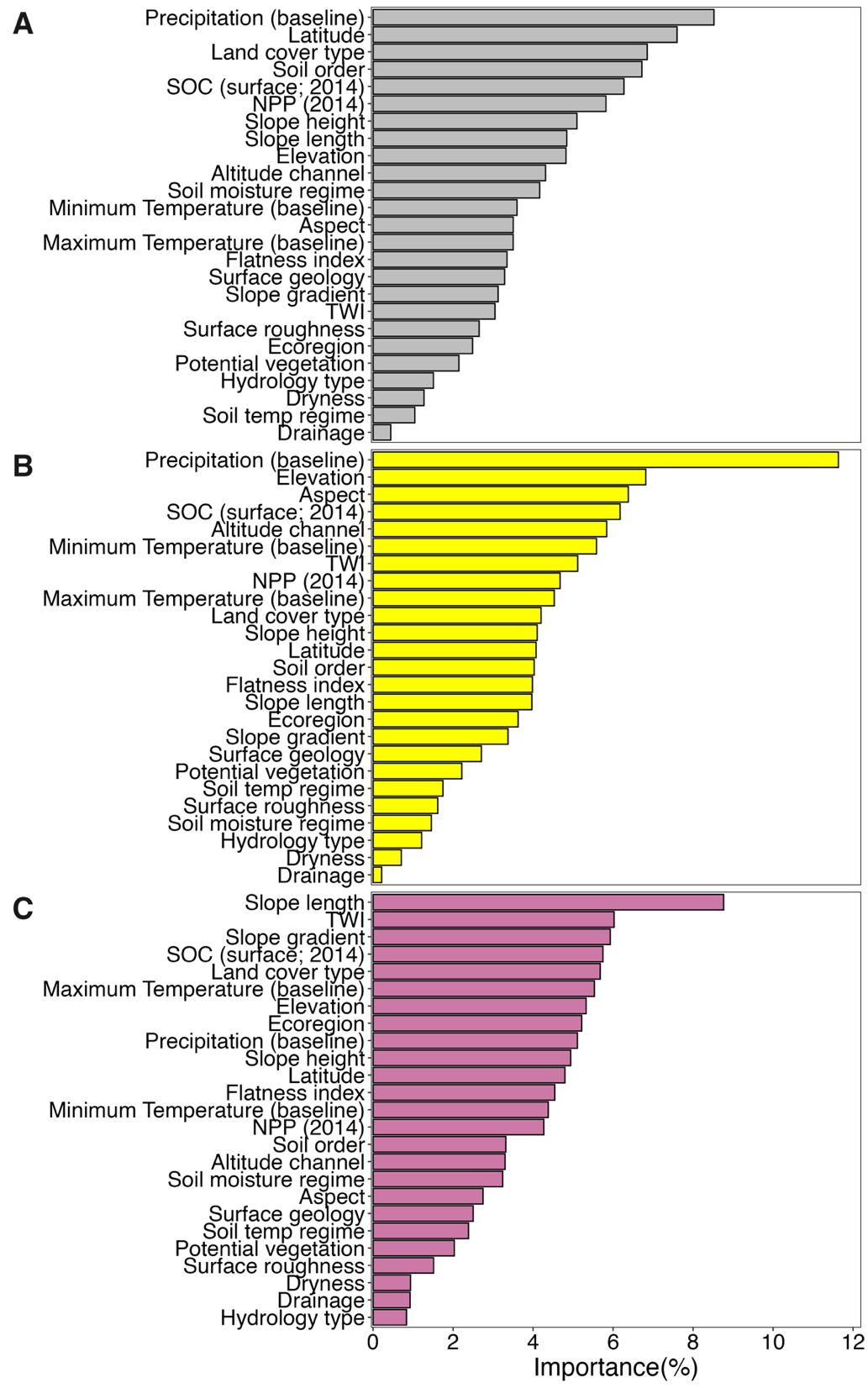


Figure S2. Importance of environmental factors in predicting soil enzyme abundance in continental US. Grey (A; C-degrading enzyme), yellow (B; N-degrading enzyme) and pink colors (C; P-degrading enzyme) indicate different enzyme categories.

Figure S3. Spatial maps of carbon (A), nitrogen (B), and phosphorous (C) degrading enzyme abundance in the baseline period (1985-2014).

Figure S4. Spatial maps of carbon, nitrogen, and phosphorous degrading enzyme abundance under the future Shared Socioeconomic Pathways (SSP) 245 (A-D) and 585 (E-H). A and E: BCC-CSM2-MR, B and F: CanESM5-CanOE, C and G: UKESM1-0-LL, D and H: CESM2.

Figure S5. Spatial maps of changes (%) in carbon, nitrogen, and phosphorous degrading enzyme abundance under the future Shared Socioeconomic Pathways (SSP) 245 (A-D) and 585 (E-H). A and E: BCC-CSM2-MR, B and F: CanESM5-CanOE, C and G: UKESM1-0-LL, D and H: CESM2.


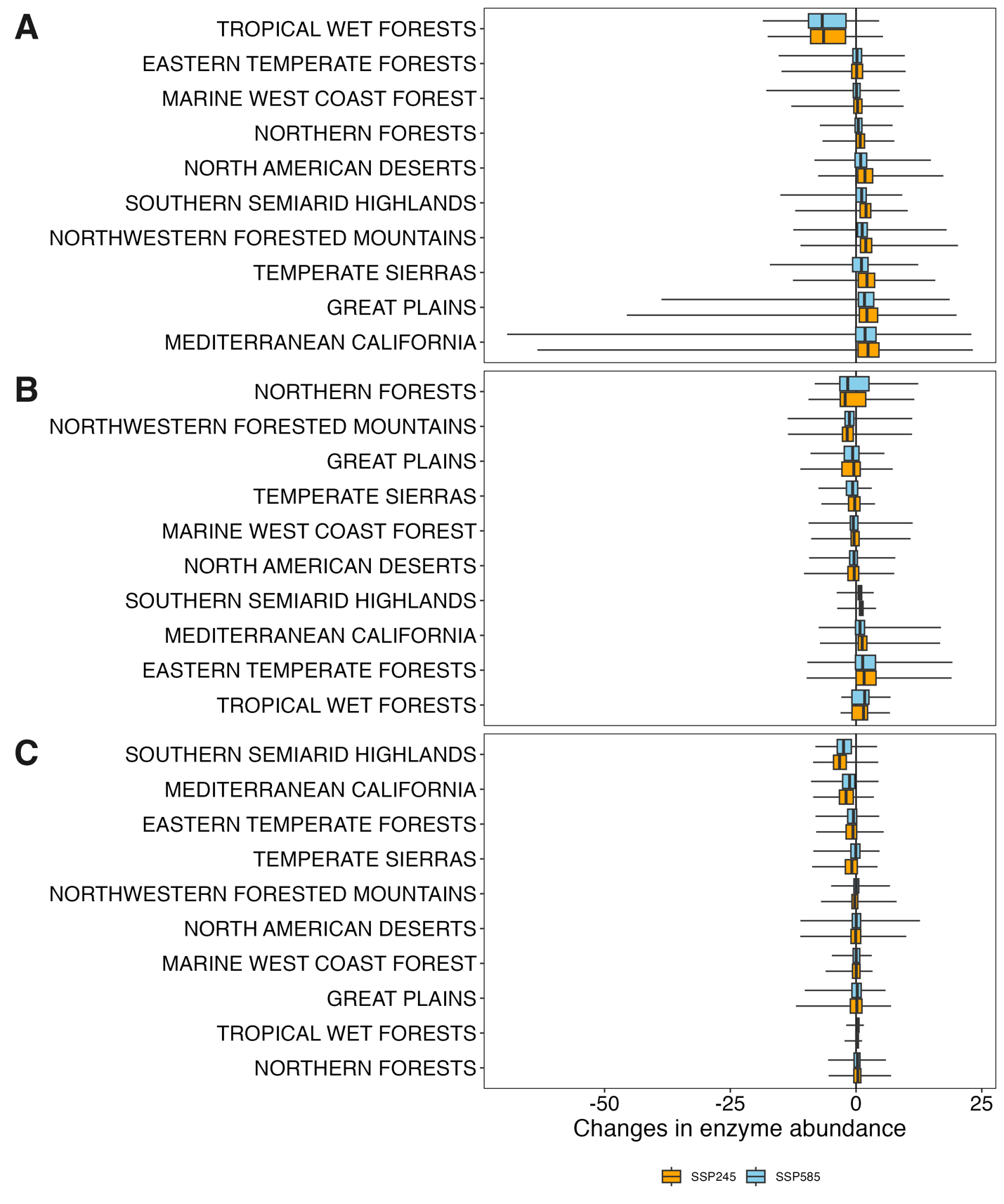


Figure S6. Changes in carbon (A), nitrogen (B), and phosphorous (C) degrading enzyme abundance due to scenarios (SSP 245, blue color; SSP 585, orange color).
